# Atypical Descending Paralysis in Miller Fisher Syndrome: A Rare Variant of Guillain-Barre Syndrome

**DOI:** 10.7759/cureus.7223

**Published:** 2020-03-09

**Authors:** Muhammad Humayoun Rashid, Hafiz Khawaja Muhammad Yasir, Muhammad Farhan Zahid, Ahmad Ali Khan, Mehjabeen Ahmad

**Affiliations:** 1 Neurology, Bakhtawar Amin Medical and Dental College, Multan, PAK; 2 Internal Medicine, Nishtar Medical University and Hospital, Multan, PAK; 3 Anatomy, Bakhtawar Amin Medical and Dental College, Multan, PAK

**Keywords:** case report, neurology, gbs, miller fisher syndrome, descending paralysis, gbs variant, symmetric paralysis, anti gq1b, plasmapheresis, internal medicine

## Abstract

Miller Fisher syndrome (MFS) is a rare variant of Guillain-Barre syndrome (GBS) which usually presents with descending paralysis. Common symptoms are ophthalmoplegia, ataxia, and areflexia. Our case presented with an atypical presentation. A 52-year old lady presented to the neurology outpatient department with frequent falls, blurring and doubling of vision and difficulty swallowing. These symptoms followed mild non-bloody diarrhea for two weeks ago. She had bilateral ptosis, lateral gaze palsy in both eyes, absent gag and cough reflex; she was unable to walk in a straight line and had right-hand grip weakness. Other motor and sensory examination were normal. She was admitted, kept under observation and investigated accordingly. Cerebrospinal fluid (CSF) analysis showed albuminocytologic dissociation. Nerve conduction studies showed slowed conduction in abducent, glossopharyngeal, vagus, and the right ulnar nerve. Blood analysis showed antiganglioside GQ1b antibodies; hence, the diagnosis of MFS, a variant of GBS, was made. Empirically plasmapheresis and then after confirmation intravenous immunoglobulins (IVIG) were used as treatment options. She recovered gradually within four weeks.

## Introduction

Guillain-Barre syndrome (GBS) is an autoimmune disorder of the peripheral nervous system characterized by weakness, usually symmetrical, and evolving over a period of several days or more [1]. After the possible elimination of poliomyelitis, GBS has become the most common cause of paralysis with an incidence of 0.5 to 2 cases per 100,000 population [2,3]. The reported incidence is influenced by the diagnostic criteria adopted as well as the thoroughness of case‐findings. GBS is thought to have at least four subtypes [4]. These subtypes are the acute motor axonal neuropathy (AMAN), acute motor and sensory axonal neuropathy (AMSAN), acute inflammatory demyelinating polyradiculoneuropathy (AIDP), and Miller Fisher syndrome (MFS). The Fisher's syndrome subtype is seen to have rare descending paralysis (not the usual ascending paralysis) and is especially associated with antibodies against ganglioside GQ1b, and similar cross-reactivity with ganglioside structures in the wall of Campylobacter jejuni [5]. Different strains of Campylobacter, as well as the host factors, are likely to play an important role in determining who develops GBS and the nerve targets for the host immune attack. Cerebrospinal fluid (CSF) evaluation demonstrates albuminocytologic dissociation in 90% of cases. AIDP was the first to be recognized over a century ago and is the most common form of GBS. Other variants such as AMNA and AMSAN have also been reported in the past few decades. But the Miller Fisher variant along with other non-categorized variants are still a rare find. Common symptoms include muscle weakness from mild to severe, limb paresthesia, autonomic symptoms, and urinary retention. Gastroparesis and diaphragm paralysis are late complications. Ophthalmoplegia and ataxia are rarest of the symptoms usually seen in the MFS subtype [6].

## Case presentation

A 52-year-old lady of normal height and build presented to the neurology outpatient department of the Nishtar Medical University and Hospital Multan. The presenting complaints of this young lady were frequent falls for one week, blurring and doubling of vision for two days, and the inability to swallow properly for two days. She said that whenever she tries to swallow anything, she ends up coughing. On inquiring further, she mentioned she had diarrhea two weeks ago, which was mild, non-bloody and relieved within three days after taking medication. She did not notice her falls earlier until the visual symptoms kicked in. She did not have these symptoms before and her family history was insignificant. She was a housewife by occupation. On physical examination, her eyelids were drooping bilaterally and abnormal lateral gaze was present in both eyes. Cough and gag reflex were absent. Visual acuity was normal bilaterally. Angle of the mouth, wrinkling of the forehead, and whistling were intact. On asking the patient to walk in a straight line, she was unable to do that. Further sensory and motor examination had no other finding except weakness of right-hand grip. The superficial and deep reflexes in both the upper and lower limbs were absolutely normal. She was breathing normally. After the detailed examination, the patient was admitted to the neurology inpatient department for the sake of keeping her under observation and investigating the real cause. All the baseline investigations including complete blood count (CBC), liver function tests (LFTs), renal function tests (RFTs), chest X-ray, erythrocyte sedimentation rate, and urinalysis were normal. The data is summarized in Table 1.

**Table 1:** Summary of all baseline reports WBC: White blood cells; RBC: Reb blood cell; HCT: Hematocrit; MCV: Mean corpuscular volume; PCV: Packed cell volume; HPF: Highest possible frequency; fl: Femtolitre.

CBC	NORMAL	PATIENT’S
WBC count (10^3/ul)	4 – 11	6.80
RBC count (10^3/ul)	4.2 – 5.4	4.89
Hemoglobin% g/dl	11	11.5-16.5
HCT (PCV) %	26 – 50	30.6
MCV (fl)	77-96	62.6
Platelets (10^3/ul)	150-400	342
Neutrophils %	40-75	40.82
Lymphocytes %	20-45	48
LIVER FUNCTION TESTS		
Bilirubin total (mg/dl)	0.1 – 1	0.6
Alanine aminotransferase (ALT) U/L	< 34	38
Aspartate aminotransferase (AST) U/L	< 35	39
Alkaline phosphatase (U/L)	46 - 122	56
Gamma glutamyl transferase (U/L)	< 38	31
RENAL FUNCTION TESTS		
Serum creatinine (mg/dl)	0.6 – 1.1	0.82
Urea (mg/dl)	10 – 50	38
Blood urea nitrogen (mg/dl)	8 - 22	18
URINE EXAMINATION		
PH	4.5 – 7.8	5.0
Sugar	Nil	Nil
Protein	Nil	Nil
Ketone	Nil	Nil
Pus cells /HPF	Nil	2 - 4
RBCs /HPF	Nil	Nil
Nitrite	Nil	Nil

Blood cultures for Campylobacter jejuni, herpes, mycoplasma, and syphilis also came out to be normal. During her stay in the hospital, she developed worsening of dysphagia, tachycardia at times and increased sweating on face. Her blood pressure and oxygen saturation remained normal throughout her stay. Computed tomography/magnetic resonance imaging (CT/MRI) of the brain was done to rule out any central nervous system pathology; the test was normal. So far, the picture was of a young lady with acute onset descending paralysis following mild diarrhea, involving cranial nerves, dysphagia, and mild right arm weakness. Later she developed autonomic symptoms and worsening dysphagia.

This overall presentation pointed towards an atypical presentation of GBS and so, plasmapheresis was planned immediately in accordance with our suspicion without waiting any further. CSF analysis was done which showed albuminocytologic dissociation (Table 2). Proteins in the CSF were 350 to 410 mg/dl and hardly few cells were found. The analysis of antiganglioside antibody in serum was positive for anti-GQ1b antibody, which are specific for MFS subtype of GBS. Nerve conduction studies were done which showed slow velocity of conduction in bilateral abducens nerves and in few branches of glossopharyngeal and vagus nerves. Conduction velocity in ulnar nerve of the right hand was also abnormal. All other nerves including radial, median, peroneal, femoral, tibial were found to have normal conduction velocities bilaterally. History data, neurological examination, results of CSF analysis, peripheral nerve conduction studies, and the additional result of positive antiganglioside anti-GQ1b antibodies helped us to reach the final diagnosis of MFS, a variant of GBS.

**Table 2:** Cerebrospinal fluid (CSF) examination WBCs: White blood cells; RBCs: Red blood cells.

CSF ANALYSIS	DAY 2	DAY 4
Color	Colorless	Colorless
Appearance	Clear	Clear
Glucose mg/dl	70	67
Protein mg/dl	356	408
WBCs /mm3	4	6
RBCs / mm3	0	0

Following her diagnosis, she was administered intravenous immunoglobulins (IVIG) in a standard dose of 0.4g/kg bodyweight for five days. She was kept under observation for a week with constant blood pressure and oxygen saturation monitoring. After the administration of IVIG, her symptoms started improving gradually. Her right-hand grip improved and she started walking on her own without any imbalance. Her visual symptoms and dysphagia became mild but were not completely recovered. She was discharged home with all the precautions and necessary treatment. She was followed up after every week for improvement of her symptoms in the outpatient department. All her symptoms improved and she recovered completely on her third follow up visit. She was counseled for future recurrences and precautions.

## Discussion

The diagnostic criteria for GBS is mentioned in Table 3.

**Table 3:** Diagnostic criteria of Guillain-Barre syndrome ALS: Amyotrophic lateral sclerosis; CSF: Cerebrospinal fluid.

DIAGNOSTIC	SUPPORTIVE	EXCLUSION
Progressive weakness of more than one limb	Cranial nerve involvement	Toxin intake
Symmetric weakness	Sensory signs and symptoms	Botulism
Hyporeflexia or Areflexia	Autonomic signs and symptoms	Poliomyelitis
Acute onset, quick progression <4 weeks	CSF protein elevation	Diphtheria
	CSF cell count < 10/mm3	ALS
	Electrophysiologic features of demyelination.	

MFS subtype of GBS is usually sudden in onset and is an autoimmune condition that develops when the immune system attacks the nervous system. The three primary symptoms of MFS are: 1) Loss of control of body movements, including weakness or uncontrolled movements 2) Loss of reflexes, particularly in the knees and ankles 3) Weakness in the face, including facial drooping, difficulty keeping the eyes open, and blurred vision [7-9] For most people, symptoms begin in the eyes. Many people with MFS struggle to walk and may waddle or walk very slowly. Some people experience other neurological symptoms, such as difficulty urinating. Researchers suggest that something, usually an infection, triggers the body to produce an antibody that leads to MFS. In our patient, infectious diarrhea could have triggered the autoantibodies production. These antibodies cause damage to the peripheral nerves, affecting the eyes, muscles, and sometimes the bladder. Among the infectious agents Campylobacter jejuni, mycoplasma, and herpes simplex are the common triggers, but many other rare infectious agents can also cause that. Diagnosis of GBS is mainly made clinically and with CSF studies to look for albuminocytologic dissociation. Imaging studies are not usually used, but can be done to rule out other illnesses such as multiple sclerosis, amyotrophic lateral sclerosis, etc. But for MFS, as the presentation is rare and resembles botulism a lot, we do specific antibody GQ1b assay in the blood which makes the diagnosis absolutely sure [10-12]. In 2001, Odaka coined the term anti-GQ1b syndrome (antiGQ1b Sy) performing a large study on 194 patients and proving the presence of anti-GQ1b antibody in MFS patients [11]. Sometimes nerve conduction studies are also done to assess the degree of demyelination. Other differential diagnosis includes stroke and polio as well, which are also investigated accordingly for exclusion. Because MFS is a variant of GBS; the treatment options for GBS and MFS are the same, one of them being plasmapheresis [13]. This means that the patient’s blood is removed from his body, cleaned, and then put back into the body. This procedure extracts all the antibodies that attack Schwann cells and cause demyelination of peripheral nerves. This procedure is time consuming. The other treatment option is IVIG [14]. These immunoglobulins competitively inhibit the autoreactive antibodies from destroying the Schwann cells. Sometimes, if the patient develops complications, then they are dealt with accordingly. For example, if respiratory paralysis develops due to phrenic nerve involvement, ICU admission and ventilator support is required. Cranial nerve involvement, most commonly facial and abducens nerve, is seen in MFS, but other cranial nerves like glossopharyngeal and vagus, as in our patient, can also be involved [15-17]. Full recovery usually takes 2-4 weeks and relapses are rarely reported clinically.

## Conclusions

Patients presenting with acute descending symmetrical paralysis and symptoms such as ophthalmoplegia, ataxia, and upper limb involvement can be considered to be suffering from MFS which is a rare variant of GBS. This suspicion should be diagnosed properly with CSF analysis, nerve conduction studies, and specific antiganglioside GQ1b antibodies. After the diagnosis, plasmapheresis and IVIG are the two options to treat this condition. Symptoms improve gradually in 2-4 weeks and relapses are rare.
